# Critical reflections on medication overuse headache in patients with migraine: An unsolved riddle in nociception

**DOI:** 10.1016/j.ynpai.2025.100179

**Published:** 2025-02-09

**Authors:** Alberto Chiarugi, Daniela Buonvicino

**Affiliations:** a Section of Clinical Pharmacology and Oncology Department of Health Sciences University of Florence Florence Italy; b Headache Center and Clinical Pharmacology Unit Careggi University Hospital Florence Italy

**Keywords:** Migraine, Medication overuse headache, CGRP, Pain chronification

## Abstract

Migraine chronification very frequently exposes patients to the inevitable risk of excessive symptomatic intake that, in turn, prompts development of medication overuse headache (MOH). The latter further compromises headache severity establishing a vicious cycle of symptomatic intake and relapsing head pain that critically worsens the overall clinical status of patients. A great deal of attention has been focused on MOH pathogenesis, and thanks to preclinical and clinical studies knowledge about this disorder is now remarkably advanced. Still, some open questions remain regarding issues related to the neurobiology and neurochemistry underpinning pain chronification in MOH patients, as well as the remedies capable of interrupting pronociceptive cephalic sensitization and drug overuse. Here, a critical reappraisal of these issues is provided in an attempt to gain deeper insight and promote debate on a pain disorder that still represents a conundrum in the field of nociception.

## Highlights

• Mechanisms by which symptomatic overuse prompts headache chronification only in patients predisposed to migraine still wait to be identified.
• Medication overuse headache (MOH) is the inevitable consequence of the need for pain relief and is not driven by compulsory disorders.
• Anti-CGRP therapies do not prompt MOH and emerge as efficacious remedies to provide long-lasting suppression of medication overuse in patients.
• Deciphering the mechanisms underlying pronociceptive cephalic sensitization is useful for understanding pain chronification in patients.

## Background

Migraine pathogenesis still waits to be unequivocally identified in spite of remarkable therapeutic achievements. Among the multiple pathophysiological events that need to be deciphered, evidence that migraine worsens if treated too often is one of the most intriguing. This condition, defined as medication overuse headache (MOH), is very well known among physicians, headache specialists, as well as patients with a history of high-frequency or chronic migraine. Given the close connection among MOH, headache chronification and refractoriness to therapy, as well as their socioeconomic burden, a great deal of effort has been focused on defining the nosological, genetic, psychological, neurochemical as well as clinical features of MOH (Ashina et al., 2023). Also, MOH preventive strategies and treatment approaches have been deeply investigated (Diener et al., 2019).

Here, we exclusively focus on key aspects of MOH pathogenesis, referring the reader to comprehensive reviews dealing with the general features of the disorder in adults (Ashina et al., 2023, Diener et al., 2019, Evers and Marziniak, 2010, Vandenbussche et al., 2018, Raggi et al., 2024, Aramruang et al., 2024), as well as in children (Frattale et al., 2024). Some preclinical and clinical aspects of MOH will be analyzed, with the aim of providing a critical reevaluation of current theories. In spite of numerous preclinical and clinical studies, various issues concerning the neurobiology and the molecular mechanisms of MOH wait to be clarified. Disproving or strengthening hypotheses on MOH pathogenesis, on the one hand might provide important neurophysiological information, and on the other might help design specific preventative and symptomatic approaches for patients. Although medication overuse promotes chronification of multiple types of headaches (Ashina et al., 2023), here we will only refer to migraine as the prototypical, predisposing factor of MOH.

## MOH in the general context of human pain disorders

According to diagnostic criteria proposed by the International Headache Society, MOH occurs in patients experiencing headache worsening (at least 15 headache days per month) concomitant to overuse for at least 3 months of triptans, opioids, ergotamine and combination analgesics (≥ 10 intakes/month), as well as paracetamol or NSAIDs (≥15 intakes/month) (Westergaard et al., 2014). Notwithstanding these strict nosological criteria, which are not always completely met when translated to the clinical setting, the key question that emerges is how symptomatic overuse operates to worsen headache severity. To answer this question, it may be relevant to first put symptomatic drug overuse into the more general context of pharmacotherapy. In particular, it is worth asking whether the pathophysiological dynamics of MOH are shared by other nociceptive disorders. The question, therefore, should be whether repetitive treatment of a given episodic, painful disease increases its frequency. In other words, is “*pain chronification by analgesic overuse”* a common pathophysiological event in pain therapy? Apparently, with the notable exception of opioid-induced pain hypersensitivity (Ossipov et al., 2005), the answer is negative. In keeping with this interpretation, repetitive treatment of osteoarthritic pain with NSAIDs does not worsen but actually improves the joint disorder. This is because overuse of pain symptomatics needs a still obscure neurophysiological substrate to establish MOH (Goadsby, 2006). For instance, in a cohort of rheumatologic patients 70 % of them overused painkillers but did not report pain worsening (Williams et al., 2008), in agreement with the notion that regular use of analgesics does not prompt MOH in non-headache patients (Bahra et al., 2003). Even more convincing is the notion that pronged exposure of cluster headache patients to daily sumatriptan injections does not promote either cluster headache chronification or a pattern of MOH. This is in spite of the fact that sumatriptan *per se* is a potent inducer of MOH when overused by migraine patients (Evers and Marziniak, 2010), and, as discussed below, its daily administration to rodents prompts a state of latent pain sensitization (De Felice et al., 2010, De Felice et al., 2010). Clinical evidence, therefore, teaches that in order for a subject to develop MOH, both symptomatic overuse and a migraine phenotype are prerequisites. More precisely, ongoing migraine is not mandatory to develop MOH, being a past history of migraine in a subject overusing analgesics for a different pain disorder sufficient to trigger MOH (Bahra et al., 2003). As outlined below, these notions are relevant when put into the context of animal models of MOH.

## MOH is not a reward-seeking disorder

Clinical experience shows that MOH patients often present patterns of severe medication overuse, with anecdotal reports of daily intake of numerous painkiller pills for years. Inevitably, the rate of drug assumption, its quantitative aspects, as well as the tendency of MOH patients to mix different drugs, suggest underlying compulsive behaviors (Calabresi and Cupini, 2005). This impression is somehow strengthened by evidence that often MOH patients are not concerned about side effects caused by the overused medications, thereby revealing apparent traits of substance use disorder. The ability of compounds present in antimigraine formulations such as barbiturates or opioids to induce drug dependence further reinforces the widely accepted idea that compulsory drug use concurs to MOH development (Saper et al., 2005, Cevoli et al., 2009, Radat et al., 2013, Takahashi et al., 2021). Remarkably, demonstrating that a drug-seeking behavior really represents a component in MOH pathogenesis would be of relevance not only to the understanding of the neurobiological underpinnings of the disorder, but also to the design of innovative detoxification paradigms to help overuse discontinuation. We claim, however, that MOH is not driven by compulsory behaviors. MOH patients, indeed, almost always feel guilty for drug overuse, revealing a clear lack of attitude toward drug abuse or psychological traits predisposing to substance use disorder. Simply put, the inability of MOH patients to control analgesic consumption is merely due to their persistent need to kill pain. In other words, drug overuse is just secondary to pathology, and not promoted by a primary, neurophysiological trait of the patient’s brain. Inevitably, each patient may have a high or low predisposition to the intake of xenobiotics that concurs to define a more or less severe MOH. Still, what drives symptomatic overuse is the need for pain relief and not drug craving. Again, clinical practice somehow confirms this view. For instance, do MOH patients who reverse to episodic migraine thanks to medication withdrawal/detoxification still seek the same medications in the absence of migraine pain? The clinic teaches that the answer is negative, but should be positive in case overuse were due to a primary craving for painkillers. Further, one may wonder whether drug overuse for chronic pain conditions such as NSAIDs for disc herniation, or morphine sulfate for intestinal adhesions should be considered compulsory, drug-seeking behavior. In this light, we must differentiate drug *craving* from drug *need*, both lead to excessive drug intake, but the former prompts *abuse* while the latter causes *overuse*. A neurochemical perspective may strengthen this interpretation. Indeed, the two drug classes most frequently overused by patients with migraine such as triptans and NSAIDs, as well as their respective targets 5HT1_B/D/F_Rs and COX1/2, do not act as dopamine-releasing agents in the reward pathway originating in the ventral tegmental area and projecting to the nucleus accumbens. Actually, 5HT1_D/F_Rs typically inhibit presynaptic release.

Three final notes may convince those still reluctant to rule out a compulsory component in MOH pathogenesis. First, although prototypical psychological traits such as emotional load and hypervigilance show comorbidity with migraine, smoking, alcohol intake, gambling or other dopamine-releasing behaviors do not. In fact, the opposite is true, i.e. patients with migraine adopt very rigid lifestyles (Seng et al., 2022). Second, at variance with what occurs during drug dependence, MOH patients tend to increase the number of intakes but not the dose at each intake. Lastly, while counseling can help MOH patients to stop overuse and maintain a “drug-free” status (Munksgaard et al., 2011, Diener et al., 2020), educational programs are not such very efficacious in substance use disorders.

Overall, patients can benefit from education (Gosalia et al., 2024), as well as from interpreting drug overuse during MOH as a disorder distinct from drug dependence. During counseling, explaining that drug overuse is an inevitable consequence common to all those individuals experiencing chronic pain can alleviate the sense of guilt for excessive symptomatic intake, improving mood and facilitating discontinuation.

## Clinical inconsistencies of MOH animal models

A great deal of effort has been focused on the development of animal models of migraine (Vuralli et al., 2019, Andreou et al., 2010, Spekker et al., 2024). Admittedly, given that migraine appears a human-specific neurological disorder, then animal models might not be so reliable, being closer to conditions of cephalic pain than to a state of deranged sensory processing. The inability to reproduce in an animal the spontaneous migraine trigger, i.e. the “brain state” of a patient with migraine (Peng and May, 2019, Altamura et al., 2021), is probably the major difficulty to model migraine in the laboratory. This shortcoming, however, appears absent in the case of preclinical models of MOH. This is because the schedules of chronic exposure to antimigraine drugs adopted by MOH patients can be easily reproduced in laboratory animals. Indeed, multiple drug overuse paradigms have been designed preclinically to model MOH pathogenesis (Ashina et al., 2023, Diener et al., 2019), and understand the induction potential of new symptomatic remedies (see below). With this concept in mind, the various paradigms of chronic exposure to antimigraine drugs adopted in experimental animals have been accepted as reliable MOH models. The majority of preclinical studies adopted treatment schedules consisting of daily exposure of rodents to triptans, opioids or NSAIDs and evaluated whether/how they alter peripheral and central pain pathways. A key finding is the ability of prolonged sumatriptan exposure to prompt pronociceptive sensitization in rats and mice, with allodynia developing in cephalic and extracephalic (paws) regions. This is paralleled by upregulation of CGRP and NO signaling in the trigeminal ganglion of both animal species (De Felice et al., 2010, De Felice et al., 2010, Bonnet et al., 2019). Although the upregulation of CGRP signaling has been considered of obvious pathogenetic relevance, recent findings in CGRP KO mice confute this hypothesis (Ryu et al., 2024). Nevertheless, the state of triptan-induced latent pain sensitization in the rat nicely recapitulates headache of MOH patients. However, if we put the experimental model *per se* and the related findings into the clinical context of MOH, some apparent inconsistencies emerge. First, the short exposure to sumatriptan adopted in this rodent MOH model (De Felice et al., 2010, De Felice et al., 2010) is not consistent with the time frame necessary to prompt MOH in patients. Indeed, cephalic allodynia in rats starts after a 6-day sumatriptan exposure with a total of 3 administrations (De Felice et al., 2010, De Felice et al., 2010, Rau et al., 2020), a temporal kinetic of symptomatic exposure non-consistent with those much more prolonged occurring in MOH patients. Even the finding that sumatriptan-induced pronociceptive sensitization spreads to peripheral structures such as the plantar surface is at odds with the clinic of MOH patients who typically complain about headache but not plantar/peripheral allodynia (unless a fibromyalgia component or neck pain (Hong et al., 2024) is present).

**Fig. 1:**
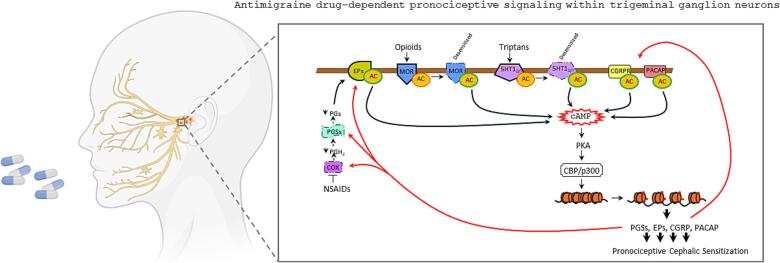
Neurochemical pathways sustaining pronociceptive cephalic sensitization within the trigeminal ganglion during MOH. Chronic exposure of trigeminal ganglion neurons to headache remedies such as triptans or opioids prompts hyperactivation of 5HT1_D/F_ and µ- (MOR) receptors with hyperactivity of adenylate cyclase (AC) and long-lasting accumulation of cAMP. Indeed, both triptans and opioids act on receptors that inhibit adenylate cyclase (red), and their overactivation leads to receptor desensitization (dotted line) with upregulation of AC activity (green line) and cAMP formation. Likewise, chronic inhibition of cyclooxygenase (COX) by NSAIDs prompts sustained reduction of PGH2 with ensuing decreases of effector prostanoids (PGE2, PGF2α and PGI2) synthesized by prostaglandin synthases (PGSs). This, in turn, leads to super-sensitivity of endo prostanoid receptors (EPs) that contribute to accumulation of cAMP. The latter boosts PKA activity and downstream activation of transcription factors endowed with histone acetyl-transferase activity such as CBP/p300. Chromatin unraveling prompted by histone acetylation (red dots) at specific promoters then triggers pronociceptive derangement of transcriptional homeostasis with upregulation of genes associated with migraine pain such as COX, PGSs, EPs, CGRP and pituitary adenylate cyclase-activating peptide (PACAP) (Buonvicino et al., 2018). These proteins, in turn, activate (red arrows) a vicious cycle of adenylate cyclase activation leading to the chronification of cephalic pain. (For interpretation of the references to colour in this figure legend, the reader is referred to the web version of this article.)

To model the temporal kinetics of MOH development more closely, our group adopted a MOH paradigm consisting of exposing rats for 30 days to eletriptan or indomethacin (Buonvicino et al., 2018). This study confirms the development of allodynia in the orofacial regions as well as in the animal forepaw. A key and somehow surprising finding provided by this study is that eletriptan and indomethacin exposure prompt an almost *identical* pronociceptive change in the transcriptome of the trigeminal ganglia of animals. Specifically, gene array analysis reveals that, in addition to CGRP and its receptor components, also genes coding for TRPV1 and −A1, substance P, endogenous opioids, 5HT1_B/D_Rs, as well as cyclooxygenases and prostaglandin synthases undergo substantial and coherent transcriptional upregulation (Buonvicino et al., 2018). The latter, according to the role played by these genes in nociception, may well build up a trigeminal ganglion proteome consistent with the latent state of pain sensitization. In apparent contrast with these findings, however, in spite of pronociceptive sensitization of the forepaw, no evidence for transcriptome changes similar to those recorded in the trigeminal ganglion has been found in the corresponding dorsal root ganglia (Buonvicino et al., 2018). Findings, therefore, somehow weaken the causative role of transcriptome derangement in the trigeminal ganglion to cephalic sensitization. Still, the almost identical changes prompted by eletriptan or indomethacin in trigeminal ganglion pronociceptive transcripts are well in keeping with the similar pattern of MOH in patients overusing triptans or NSAIDs (Oh et al., 2022). From a pathophysiological perspective, the ability of drugs with completely different pharmacodynamics to similarly affect trigeminal ganglion gene expression profiles suggests the existence of a common denominator downstream from 5HT1_B/D/F_ and prostaglandin receptors that plays a pivotal role in rat, and possibly human, pain sensitization. In light of the transduction pathways involved, such a common agent might be cAMP (see Fig. 1). It is reasonable that signaling homeostasis at trigeminovascular terminals (both peripheral and central) becomes dysfunctional following persistent, triptan-dependent inhibition of adenylate cyclase. Chronic agonism on 5HT1_B/D/F_ receptors can prompt receptor desensitization that, in turn, may lead to increased cAMP signaling. A similar derangement of cAMP homeostasis at trigeminal terminals can be induced by NSAIDs that, under chronic exposure paradigm and prostanoid synthesis suppression, may prompt prostaglandin receptor upregulation, thereby sensitizing to adenylate cyclase-activating PGD2, PGE2 as well as PGI2. Notably, sustained derangement of cAMP signaling alters CREB transcription factor activity that, upon phosphorylation by PKA, binds to cAMP-responsive elements at gene promoters recruiting CBP/p300 within transcriptional supramolecular complexes (Breen and Mapp, 2018). CBP/p300, in turn, thanks to its acetyltransferase activity, epigenetically modifies chromatin architecture as well as trigeminal ganglion gene expression profiles (Mitsikostas et al., 2011, Ramachandran et al., 2018) (see Fig. 1). In keeping with a key role of acetylation-dependent, epigenetic rearrangement in MOH pathogenesis, the two potent histone deacetylase inhibitors (HDACis) givinostat and panobinostat counteract trigeminal ganglion overexpression of CGRP and its receptor subunit RAMP1 in rats chronically (30 days) exposed to eletriptan, also reducing their pronociceptive sensitization (Urru et al., 2022). These epigenetic effects correlate with reduced capsaicin-dependent vasodilatation (a proxy of CGRP release) in the trigeminal territory, diminished photophobia, as well as reduced periorbital allodynia in rats chronically exposed to eletriptan (Urru et al., 2022). Surprisingly, this study shows that neither chronic exposure to eletriptan, nor concomitant challenge with HDACis affects transcriptional homeostasis of CGRP and its receptor components within the trigeminal nucleus (Urru et al., 2022). These findings taken together on the one hand corroborate the role of transcriptional deregulation in the pathogenesis of MOH, and on the other identify epigenetic modulators as drugs with a therapeutic potential in detoxification of MOH patients (see below). In this regard, the notion that valproic acid is a HDAC inhibitor (Monti et al., 2009) further strengthens the hypothesis that epigenetic remodeling at specific gene promoters triggers chronification of migraine pain. The association of a genetic variant of HDAC3 with the severity of symptomatic overuse in MOH patients (Pisanu et al., 2015) also suggests that specific epigenetic traits predispose to excessive antimigraine symptomatic intake. Finally, although still debated (Woldeamanuel et al., 2015, Kaltseis et al., 2022), the ability of corticosteroids to promote symptomatic detoxification in MOH patients, along with their potent and widespread epigenetic effects further corroborate the hypothesis that epigenetic treatments reset cephalic pain thresholds. Interestingly, although corticosteroids inhibit prostanoid synthesis like NSAIDs and can be adopted as acute antimigraine drugs (Woldeamanuel et al., 2015), they do not prompt MOH when, for instance, are chronically administered to patients with migraine for anti-inflammatory/immunosuppressive purposes. Studies on how steroids affect both peripheral and central nociception homeostasis might further our understanding of MOH pathogenesis, also providing information on the enigmatic mechanisms through which they operate in detoxification protocols in MOH patients.

Notwithstanding the still open, central question about the molecular mechanisms underlying derangement of trigeminovascular transcriptome in preclinical models of MOH, it is important to put these experimental findings into the clinical context in order to appreciate their relevance to MOH. As mentioned above, clinical evidence teaches that MOH does not occur in non-migraine patients overusing analgesics, being migraine, or predisposition to migraine essential prerequisites to undergo headache chronification upon symptomatic overuse (Bahra et al., 2003). The question then arises as to whether/how a pronociceptive cephalic and peripheral sensitization by symptomatic overuse in otherwise non-migraineur rodents should represent a reliable MOH model. The answer might be that latent pain sensitization by migraine symptomatics in lab animals does not recapitulate MOH simply because the sole symptomatic overuse in healthy subjects not predisposed to migraine does not prompt MOH. Still, the ability of antimigraine drugs to prompt latent cephalic pain sensitization in animals and MOH in patients with migraine is extremely intriguing and suggests common pathophysiological mechanisms underpinning derangement of nociception homeostasis. Rapid sensitization to pain in experimental animals after repeated challenges with analgesics might just be an allostatic response aimed at resetting their pain thresholds to readily detect and cope with external threats. As a provocative hypothesis, it might be speculated that molecular mechanisms responsible for pain sensitization in rodents exposed to analgesics might have been lost during evolution, but tend to reemerge in patients with migraine during MOH. In this light, MOH might be a sort of a remnant of an ancestral physiological response suppressed in humans during evolution but reawakening in patients with migraine because of their peculiar derangement of sensory processing (Goadsby et al., 2017). This reasoning should be put into the context of two fascinating, and only in part opposing, theories on the evolutionary meaning of migraine and pain chronification. Indeed, if on the one hand migraine chronification might represent a maladaptive response to repeated, defensive sickness behavior episodes (Pensato et al., 2023), on the other pain chronification via a maladaptive response to nociception might confer a survival advantage (Walters et al., 2023). Possibly, a holistic approach that puts the neurobiological basis of pain chronification into the context of Darwinian selection (Pensato et al., 2023) should help deciphering MOH pathogenesis.

Overall, when experimental findings are translated to the context of MOH patients, preclinical evidence is somehow non-consistent with the clinics. Clarifying the reasons for this apparent inconsistency will help in reaching a thorough understanding of MOH pathogenesis that might assist the development of innovative MOH preventatives, as well as detoxifying protocols.

## CGRP-targeted medications in MOH management: Undeciphered pathophysiological hints

Anti-CGRP therapies (i.e. anti-CGRP mAbs and the CGRP receptor antagonists gepants) differentiate from previous antimigraine medications in terms of both induction risk and management of MOH. Remarkably, anti-CGRP therapies not only do not prompt MOH, but emerge as efficacious tools to provide long-lasting suppression of medication overuse in patients, also showing low incidence of relapse (Pensato et al., 2022, Oliveira et al., 2024, Scheffler et al., 2024, Kong et al., 2024). According to real-world studies, anti-CGRP monoclonals erenumab, galcanezumab and fremanezumab prompt equal and stable interruption of drug overuse in up to 57–64 % (Pensato et al., 2022, Russo et al., 2020); 29–88 % (Krymchantowski et al., 2023) or 89–60 % (Scheffler et al., 2024) of MOH patients. Somehow surprisingly, anti-CGRP monoclonals show a tendency to improve chronic migraine better in the presence than in the absence of medication overuse (Scheffler et al., 2024). Even more intriguingly, these biologics improve MOH even in the absence of drug withdrawal (Scheffler et al., 2024). Emerging clinical evidence suggests that also prolonged CGRPR antagonism with gepants does not increase symptomatic intake (Oliveira et al., 2024) but, actually, counteracts MOH over time even without specific guidance for acute medication withdrawal (Goadsby et al., 2024). This is in keeping with preclinical findings showing that repeated exposure to olcegepant (Saengjaroentham et al., 2020) or ubrogepant (Navratilova et al., 2020) does not prompt allodynia in the hind paw or orofacial region of mice and rats. Consistent with this, and at variance with sumatriptan, prolonged exposure to olcegepant increases neither CGRP expression levels within central trigeminal projections, nor activity (c-Fos expression) of neurons of the trigeminal nucleus of mice (Saengjaroentham et al., 2020).

These preclinical and clinical findings suggest that the ability of anti-CGRP remedies to target trigeminal nociception downstream (i.e. post-synaptically) from the site of action of classic antimigraine remedies underpin their ability to prevent MOH induction. This interpretation, however, is somehow at odds with principles of synaptic plasticity. Indeed, one may speculate that CGRP-targeting drugs should prompt CGRP receptor upregulation and/or increased CGRP synthesis and release. These plastic changes should then sensitize to trigeminovascular nociception and promote MOH. In other words, at variance with clinical evidence, given the role of CGRP in inducing and sustaining latent cephalic sensitization, chronic therapy with anti-CGRP medications should be more prone to prompt and stabilize MOH among antimigraine remedies. Admittedly, the most obvious reason that may explain this apparent conundrum is that the role of CGRP in migraine pathogenesis and chronification is more complex than currently envisaged. It has also been proposed that the inability of anti-CGRP remedies to prompt MOH may be ascribed to their post-synaptic site of action (Saengjaroentham et al., 2020). While triptans likely promote pronociceptive sensitization via presynaptic 5HT1_D/F_ receptors at trigeminovascular terminals (Ashina et al., 2023, Diener et al., 2019), anti-CGRP remedies should exclusively impact the post-synaptic side, although the presence of presynaptic CGRP receptors at trigeminal terminals has been reported (Storer et al., 2004, Gupta et al., 2010). Still, whether and why chronic suppression of postsynaptic CGRP receptor activation, either by direct receptor antagonism or by CGRP scavenging, does not trigger synaptic plasticity waits to be understood. Lack of cephalic pronociceptive sensitization by anti-CGRP remedies, however, is also supported by the general absence of wearing-off phenomena in patients on anti-CGRP monoclonals (Asawavichienjinda et al., 2023, Florescu et al., 2024). Similarly, evidence that abrupt migraine rebound does not occur upon antibody discontinuation (Vernieri et al., 2021) also indicates that prolonged exposure to anti-CGRP medications does not sensitize the trigeminovascular system to CGRP neurotransmission. Real-world data on abrupt interruption of migraine prophylaxis with gepants will help clarify the issue of CGRP receptor plasticity upon prolonged receptor inhibition. On a theoretical basis, should it turn out that trigeminal CGRP receptors, known to be located at Aδ terminals, do not undergo plasticity, then pronociceptive sensitization during MOH should be exclusively ascribed to the presynaptic, CGRP-releasing apparatus located at C-fiber terminals. Recent findings with the antimigraine, 5HT1_F_ agonists ditans corroborate the pathophysiological role of C-fibers in MOH. In preclinical MOH models, indeed, repeated exposure to ditans such as lasmiditan (Rau et al., 2020) or its structural analog LY344864 (Saengjaroentham et al., 2020) prompt severe allodynia in mice and rats, robustly reducing their periorbital and hind paw mechanical withdrawal thresholds. Notably, onset, severity, and duration of allodynia displayed by animals exposed to the two ditans are identical to those occurring in rodents challenged with sumatriptan (Rau et al., 2020). In light of the expression of 5HT1_F_ receptors at meningeal CGRP-releasing C-fiber trigeminal terminals (Mitsikostas et al., 2023), these findings strengthen the relevance of this presynaptic component to MOH pathogenesis. It is also worth mentioning that the identical pronociceptive sensitization prompted by sumatriptan and lasmiditan (Rau et al., 2020) suggests a key role of 5HT1_F_ rather than 5HT1_D_ or 5HT1_B_ receptors in triptan-induced MOH. Again, real-world evidence will clarify the actual MOH induction potential of ditans, furthering our understanding of the molecular machinery underpinning cephalic pain sensitization by antimigraine drug overuse.

## Conclusions

MOH represents a unique clinical entity in the field of pain (Goadsby, 2006). Numerous studies focused on molecular mechanisms underlying its pathophysiology, including gut dysbiosis (Vuralli et al., 2024), and key information has now been gathered regarding genes whose expression deregulation contributes to pain sensitization in animal models. Among the multiple issues that still need to be addressed in the understanding of MOH pathogenesis, two appear of paramount importance. The first is why antimigraine drug overuse prompts MOH exclusively in patients with migraine. The second relates to the detailed deciphering of the molecular mechanisms that from chronic agonism/antagonism of receptors involved in trigeminovascular pain trigger pronociceptive cephalic sensitization. Possibly, these two open questions share the same answer that hides in the mystery of the migraine brain.

## CRediT authorship contribution statement

**Alberto Chiarugi:** Writing – original draft. **Daniela Buonvicino:** Writing – original draft.

## Declaration of competing interest

The authors declare that they have no known competing financial interests or personal relationships that could have appeared to influence the work reported in this paper.

## Data Availability

No data was used for the research described in the article.
